# Idiopathic Pulmonary Fibrosis Comorbid With Mediastinal Small Cell Carcinoma: A Clinical Dilemma

**DOI:** 10.7759/cureus.53578

**Published:** 2024-02-04

**Authors:** Muhammad Hassan Shakir, Salman Abdul Basit, Barun K Ray, Syed Muhammad Hussain Zaidi, Taibah Chaudhary, Mohammad Asim Amjad

**Affiliations:** 1 Internal Medicine, Shifa Tameer-E-Millat University Shifa College of Medicine, Islamabad, PAK; 2 Internal Medicine, The Wright Center for Graduate Medical Education, Scranton, USA

**Keywords:** rare, antifibrotic, small cell, mediastinum, mediastinal, idiopathic pulmonary fibrosis

## Abstract

We present an interesting case of mediastinal small cell carcinoma (MSCC), an exceedingly rare entity, comorbid with idiopathic pulmonary fibrosis (IPF). A 66-year-old female was first seen in the pulmonology office for abnormal chest computed tomography (CT) findings of right apical bronchiectasis and subpleural fibrotic changes with focal pleural thickening along the fissures, along with a right lower lobe nodule. Pulmonary function testing (PFT) showed an obstructive pattern with modest bronchodilator response, although subsequent PFT showed a worsening restrictive pattern with a worsening DLCO. On a follow-up CT one year later, a soft tissue density with peripheral calcification was found in the anterior mediastinum, later found to be hypermetabolic on a PET scan. Radiographically, fibrosis worsened with the appearance of worsening diffuse bilateral coarse reticular interstitial changes with lower lobe predominance, honeycombing, and areas of ground-glass opacity. A biopsy of the mediastinal lesion showed a high-grade neuroendocrine tumor. Cam5.2, insulinoma-associated protein-1, synaptophysin, and thyroid transcription factor-1 immunostains were positive. She underwent four cycles of chemotherapy with cisplatin and etoposide with a total of 60 Gy of radiation. Mediastinal mass started to decrease in size. Her respiratory status, imaging, and PFTs continued to show evidence of IPF progression. Prednisone resulted in modest clinical and radiographic response. Steroid-sparing therapy with mycophenolate mofetil, although effective, had to be discontinued due to GI bleeding. Anti-fibrotic therapy was deferred due to evidence showing a lack of clinical improvement. We discuss the existing evidence available on IPF management and proceed to highlight the deficiencies in existing data available on the management of IPF and MSCC in these patients. Most of the cases of MSCC reported in the past have managed MSCC using guidance from treatment practices for small cell lung cancer. No reported cases discuss or describe the management of IPF and MSCC in the very rare cohort of patients our case represents.

## Introduction

Small cell carcinoma (SCC) is a neuroendocrine tumor (NET), and as per the 2022 World Health Organization (WHO) classification of neuroendocrine neoplasms, it is a poorly differentiated epithelial neuroendocrine neoplasm [1]. Of note, the 2022 classification also introduces a new chapter on neuroendocrine neoplasms in non-endocrine organs. Furthermore, the WHO classifies NETs of the lung, pleura, thymus, and heart as typical and atypical NETs, large cell neuroendocrine carcinoma, and SCC [2]. SCC is a distinct histological entity characterized by small blue cells with scant cytoplasm, fine, granular chromatin, and absent nucleoli [3]. Cells grown in sheets or rosettes. A good quality histology specimen with adequate hematoxylin and eosin staining continues to be a reliable tool for histological diagnosis, although immunohistochemistry may be required to differentiate it from other NETs [3]. Pathological diagnosis of small cell lung cancers (SCLCs) is made according to the WHO classification, which classifies SCLC as a neuroendocrine carcinoma [4]. Up to 95% of SCC occurs primarily in the lung, with the remaining 5% constituting extrapulmonary small cell cancer (EPSCC) [3]. SCLC and EPSCC have similar clinical/biological behavior and are invasive tumors with early aggressive metastasis [3]. Napsin A and p40 (or p63) are negative in SCC and can be used to differentiate SCC from adenocarcinoma and squamous cell carcinoma, respectively. Neuroendocrine markers are usually positive in SCC, although they can be positive in other NETs and a small number of non-small cell lung cancer [3].

EPSCC is a rare and distinct entity and has been documented in multiple organs [5]. EPSCCs represent 2-5% of all SCCs and 0.1-0.4% of all tumors [5]. The histological features of EPSCC are similar to those of SCLC. The occurrence of SCC in non-endocrine sites is thought to be due to tumor origins from pluripotent stem cells or the development of neuroendocrine features in more typical carcinomas [5]. The disease stage for EPSCC appears to be a stronger predictor of outcomes than in SCLC [5]. The exact epidemiology and organ-specific distribution of EPSCC are not known due to the rarity of this presentation. An analysis of the National Cancer Database showed the most common site to be genitourinary organs, with the breast being the least common [6].

Idiopathic pulmonary fibrosis (IPF) is an idiopathic interstitial pneumonia (IIP) and is a progressive, chronic, fibrosing, restrictive lung disease. The 2013 American Thoracic Society/European Respiratory Society (ATS/ERS) classifies IPF as chronic fibrosing IIP [7]. The ATS/ERS also guides the diagnosis of IPF, which is based on a combination of high-resolution computed tomography (HRCT) and histological features [8]. The classical radiographic appearance of IPF is usual interstitial pneumonia (UIP). HRCT findings are reported as UIP, probable UIP, indeterminate for UIP, and CT findings suggestive of an alternate diagnosis.

Mediastinal small cell carcinoma (MSCC) is an even rarer entity, with only a few reported cases. We report a case of MSCC comorbid with IPF. We also present a brief review of the existing literature on MSCC and discuss the complexity of the management of MSCC in patients with IPF. The abstract for this case was presented at the American College of Chest Physicians (ACCP) CHEST 2023 Conference in Hawaii in October 2023.

## Case presentation

The patient is a 66-year-old female who first presented to the pulmonology office for evaluation of progressive cough and shortness of breath. Her shortness of breath had been worsening for the past six months. Her activity was limited to three to four blocks. She denied any orthopnea or paroxysmal nocturnal dyspnea or any exertional chest pain. She did report an associated dry cough. There was no hemoptysis. She denied any recent weight changes but did report associated chronic fatigue. A review of systems was otherwise unremarkable. She had a past medical history of diabetes mellitus, hypertension, hyperlipidemia, coronary artery disease with a history of percutaneous coronary intervention, tobacco use disorder with a 49-pack-year smoking history, and chronic obstructive pulmonary disease. She denied any new pets at home. She denied any significant occupational exposures. She did not have any family history of chronic lung diseases. She had had pulmonary function testing (PFT) more than one year ago with an obstructive pattern (forced expiratory volume (FEV1) 77% predicted, FEV1/forced vital capacity (FVC) 75% predicted, no significant bronchodilator response, total leukocyte count (TLC) 94%, uncorrected diffusing capacity for carbon monoxide (DLCO) 47% predicted). She also had a history of breast cancer treated with surgical excision and radiation about seven years ago. Upon initial examination, she was hemodynamically stable, saturating at 97% on room air. Her chest examination did reveal faint inspiratory crackles in the bases bilaterally.

**Figure 1:**
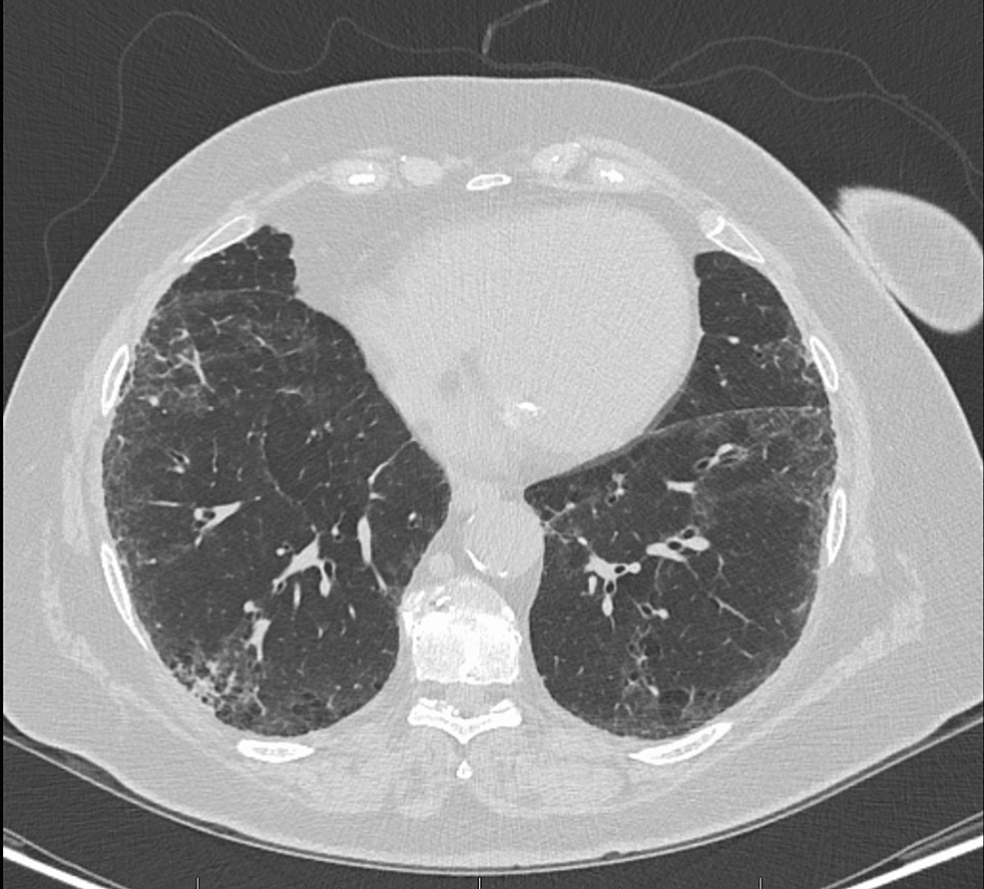
Chest CT showing evidence of lung fibrosis with subpleural fibrotic changes, peripheral honeycombing, and small areas of traction bronchiectasis

**Figure 2:**
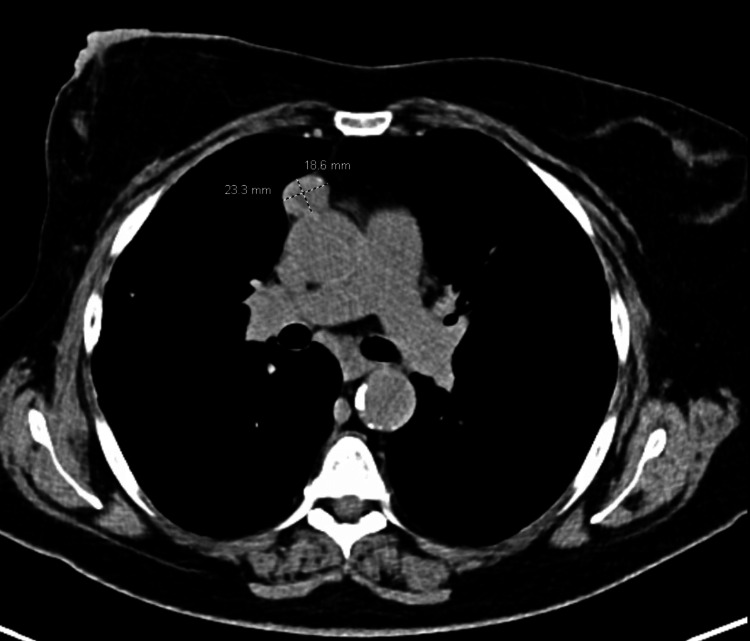
CT chest showing prevascular mass anterior to the ascending aorta measuring 1.9 X 2.3 cm

At this time, a chest CT was performed and showed diffuse bilateral coarse reticular interstitial changes with subpleural and lower lobe predominance, honeycombing, and small areas of ground-glass opacity (Figure 1). CT also showed areas of traction bronchiectasis in the right upper lobe. Findings were consistent with pulmonary fibrosis, a UIP pattern. After a multidisciplinary discussion (MDD), a diagnosis of IPF was established. CT also showed a soft tissue mass anterior to the ascending aorta measuring 1.9 X 2.3 cm (Figure 2). PFT at this time showed new restriction with impaired diffusion (FEV1 68%, FEV1/FVC 73%, no bronchodilator response, TLC 72%, and uncorrected DLCO 52% predicted). Rheumatologic workup, including rheumatoid factor, anti-cyclic citrullinated peptide, antinuclear antibody titer, anti-synthetase antibodies (e.g., Jo-1), creatine kinase and aldolase, Sjogren's antibodies (SS-A, SS-B), and scleroderma antibodies (scl-70, PM-1) was done and was non-contributory. Bronchoalveolar lavage was done, which ruled out hypersensitivity pneumonitis. The patient declined surgical or transbronchial lung biopsy. After a MDD, the patient was classified as IPF and was started on mycophenolate mofetil (MMF), which did show radiographic improvement although it was later discontinued due to an episode of lower GI bleed. She was then started on daily low-dose prednisone.

**Figure 3:**
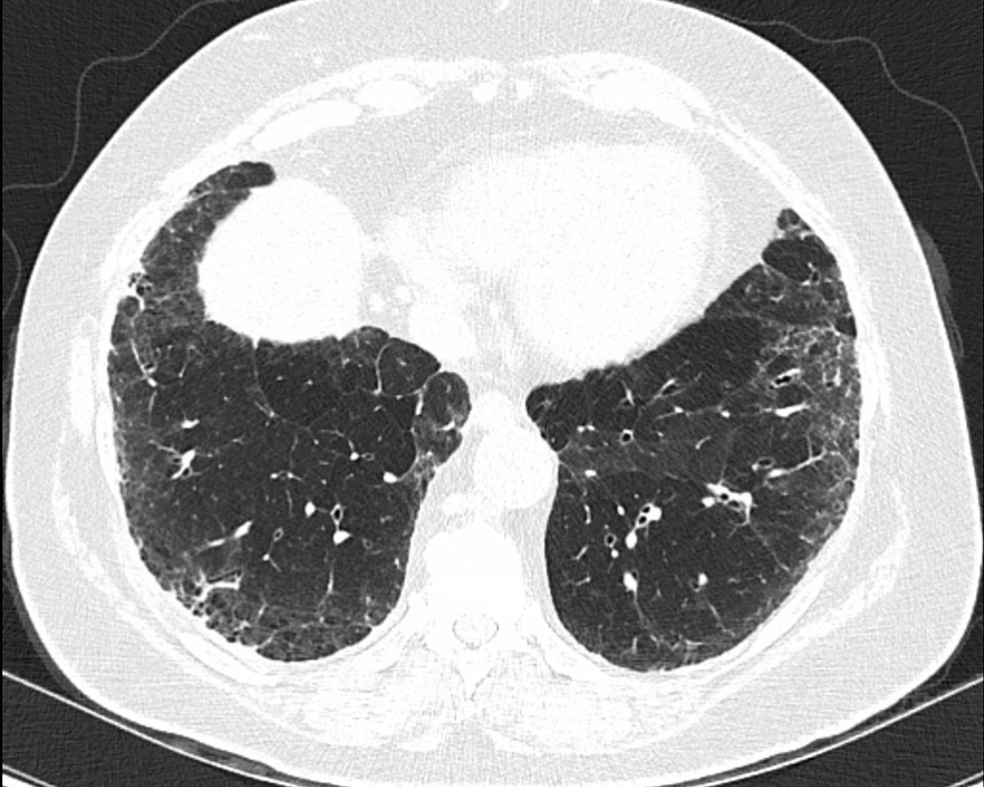
CT Chest showing worsening reticular changes, honeycombing, and traction bronchiectasis in the lower lobes

**Figure 4:**
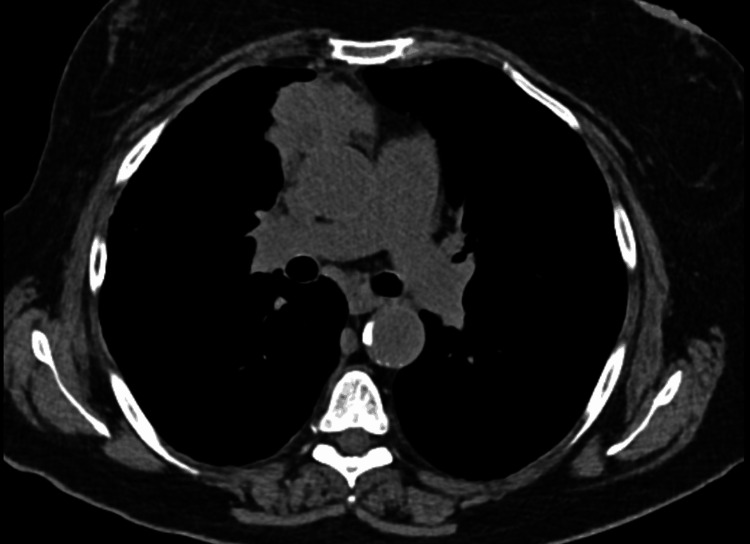
CT chest showing a large increase in the size of anterior mediastinal mass

On a follow-up appointment five months later, the mediastinal mass was static in size (1.8 cm by 2.3 cm). CT also showed worsening diffuse bilateral coarse reticular interstitial changes with lower lobe predominance, honeycombing, and small areas of ground-glass opacity (Figure 3). For monitoring of the mediastinal mass, a follow-up CT chest was arranged in three months and showed a rapid increase in the size of the mediastinal mass, this time to 4.5 cm by 7 cm (Figure 4). A PET/CT scan was performed and showed the mediastinal mass to be partially necrotic. CT-guided biopsy of the mediastinal lesion showed a high-grade NET. Cam5.2, insulinoma-associated protein-1, synaptophysin, and thyroid transcription factor-1 immunostains were positive. MIB-1 immunostain showed a proliferative index of approximately 50% in the limited material. CD3, CD20, and GATA-3 immunostains were negative. MRI brain was done which ruled out brain metastases. A PET scan was also done which ruled out any distant metastases. The patient was started on chemotherapy with cisplatin and etoposide. Intensity-modulated radiation therapy was added after cycle two of chemotherapy. The total radiation dose was 60 Gy. Follow-up CT scan showed an interval decrease in the size of the mass in the anterior mediastinum abutting the ascending aorta. Repeat PFT was done which showed relatively stable restriction and reduced DLCO (FEV1 73%, FEV1/FVC 79% predicted, TLC 70%, and uncorrected DLCO 43% predicted). An extensive goal-of-care discussion with the patient was held including a decision regarding the initiation of antifibrotics, and the patient decided against the use of the same.

## Discussion

HRCT findings are classified as either usual UIP, probable UIP, indeterminate for UIP, or CT findings suggestive of an alternate diagnosis. UIP includes all four reticular abnormalities, honeycombing, subpleural predominance, and interlobular septal thickening [8]. In our patient, the presence of ground glass opacities (GGOs) did not classify as inconsistent with UIP since the GGOs were not more extensive than honeycombing. However, the lack of interlobular septal thickening did classify our case as probable UIP. As per the guidance, probable UIP can be classified as IPF if it is agreed upon in MMD discussions. Other identifiable causes of ILD/IIP were effectively ruled out to establish a diagnosis. Interestingly, a probable UIP pattern still reflects a diagnostic confidence level of more than 70% and up to 89% [8]. HRCT findings indeterminate for UIP warrant a histological confirmation, and the ATS/ERS/Japanese Respiratory Society (JRS)/Latin American Thoracic Association (ALAT) joint statement accepts transbronchial lung cryobiopsy as a substitute for surgical lung biopsy (SLB). Pirfenidone and nintedanib are the only two approved treatments, with steroids being used for acute exacerbations [8]. The INPULSIS-1 and INPULSIS-2 trials concluded that nintedanib slows the decline of FVC and slows disease progression in IPF [9]. Pirfenidone, as per the A Study of Cardiovascular Events iN Diabetes (ASCEND) trial, has the additional advantage of improving progression-free survival and exercise tolerance [10]. One limitation of the INPULSIS trials and the ASCEND trials was the exclusion of patients with DLCO less than 30% of predicted and FVC less than 50% of predicted. Progressive pulmonary fibrosis (PPF) is defined as two of the following three: worsening respiratory symptoms, physiologic evidence of disease progression, and radiographic evidence of disease progression. Our patient did have worsening symptoms and radiographic disease progression. There is a paucity of data on the efficacy of antifibrotic medications in the management of PPF, and therapy has to be individualized to the patient and to the primary ILD, since PPF is not specific to IPF [11]. Due to the risks associated with long-term steroid therapy, immunosuppressives may be preferred in some cases, although MMF had to be discontinued in our case due to GI bleeding [11].

MSCC is a rare malignancy, with very few reported cases. Most of the reported cases are of thymic/parenchymal origin. Like all mediastinal masses, they can have a multitude of presentations. Patients can present with worsening shortness of breath, weight loss, fatigue, superior vena cava-like syndrome, and/or refractory shock due to compression of mediastinal vessels. In one of the very few reported case series of 25 cases, none of the cases presented with paraneoplastic syndromes. About 20 patients were male and 5 were female, with a mean age of 59 years. Also, 10 of these were in the anterior mediastinum, as in our case. Chromogranin A and synaptophysin were positive in 64% and 76% of cases, respectively. Various techniques, both open and closed, were used to obtain a pathology specimen. About 5% survival was 8.4%, although the median survival time varied from 23 months in limited disease to 8 months in extensive disease [12]. Diagnosis is based on histological findings augmented by immunohistochemistry. Non-surgical diagnosis raises the uncertainty between thymic and isolated anterior mediastinal NETs, and detailed histopathological evaluation of a surgical specimen is the only reliable means to differentiate between the two [12]. Reported cases have relied on treatment guidelines for the management of SCLC [13,14]. In our patient, the diagnosis was based on CT guided core needle biopsy. No thymic tissue was found in the tumor. IPF comorbid with MSCC is a unique clinical presentation not reported previously and presents several practical challenges. First, the surgical biopsy of the mediastinal mass may be limited by the functional and respiratory status of a patient with advanced IPF/PPF. Second, while the co-occurrence of IPF with SCLS is rare, the association of IPF with MSCC is even rarer and almost unheard of. There are no widely accepted treatment guidelines for SCLC in patients with IPF, which is important since most of the reported cases of MSCC treated MSCC using guidance from the treatment of SCLC. Treatment for SCLE has evolved in the past two decades, although evolving therapies do not as of yet have established safety in patients with ILD/IPF. Case reports exist of chemotherapeutic regimens used with antifibrotics in this cohort, although efficacy and safety have not been validated by prospective studies [15].

## Conclusions

In conclusion, MSCC in comorbid with IPF represents a rare presentation. While the diagnostic algorithms for IPF have evolved over the years, definitive diagnosis of a non-thymic origin MSCC requires surgical removal and microscopic analysis. It may be that at least some cases of IPF comorbid with MSCC may have been left undiagnosed due to the progressive nature of IPF precluding surgical biopsy, leaving little to no data to form an evidence basis for the management of these cases. Most of the cases reported in the past have derived guidance from management practices used for SCLC. SCLC can be comorbid with IPF, which is a rare presentation in and of its own. At present, there are no retrospective studies evaluating the safety and efficacy outcomes of available treatment modalities in this cohort of patients.
